# Toward a vestibular contribution to social cognition

**DOI:** 10.3389/fnint.2014.00016

**Published:** 2014-02-14

**Authors:** Diane Deroualle, Christophe Lopez

**Affiliations:** Laboratoire de Neurosciences Intégratives et Adaptatives, UMR 7260, Centre Saint Charles, Fédération de Recherche 3C, Centre National de la Recherche Scientifique, Aix-Marseille UniversitéMarseille, France

**Keywords:** vestibular system, social cognition, perspective taking, body ownership, mirror neurons

Social cognition encompasses perception of self and others as well as self-other interactions. Self-other interactions rely on a wide range of cognitive processes such as memory, language, reasoning, and emotion processing (Beer and Ochsner, 2006). Within the last few years, one productive line of research in social neuroscience has investigated the multisensory and motor foundations of self-other interactions, including emotion perception, emotional contagion, empathy, self-other distinction, or self-other knowledge (e.g., Singer et al., 2004; Iacoboni et al., 2005; Ambrosini et al., 2013; Manera et al., 2013). While a strong emphasis has been traditionally put on visual processes, recent research has noted that self-other distinction and mirroring also require processing of auditory (e.g., self-other voice recognition), proprioceptive, and interoceptive signals (Damasio, 2000; Schutz-Bosbach et al., 2006; Tsakiris et al., 2011; Seth, 2013; Xu et al., 2013). In spite of this multisensory development, a vestibular contribution to the embodied mechanisms of social interactions has until now been largely overlooked. This is surprising as the vestibular system has been involved in a growing number of cognitive functions (Smith et al., 2005; Miller and Ngo, 2007; Gurvich et al., 2013), in addition to its crucial role in distinguishing self- and non-self motion. The claim of the present opinion article is that vestibular information should not be ignored when investigating the sensorimotor foundations of social cognition. We present several lines of evidence indicating that vestibular signals may be involved in the sensory bases of self-other distinction and mirroring, emotion perception and perspective taking.

## Distinguishing self and non-self

One characteristic of the vestibular system is to code *absolute* body motion in space (Berthoz, 2000). The vestibular system contains “inertial sensors” (i.e., types of accelerometers and gyroscopes) activated by gravito-inertial forces generated by self-motion. As a consequence, vestibular sensors function without external references (besides Earth's gravity), i.e., without allocentric or egocentric references, in contrast with the visual and somatosensory coding of motion. Vestibular signals should be a crucial component of self-other distinction by discriminating between “*I*” (the subject of experience) have moved (or “*I*” have been moved), *another person* has moved, or the *environment* has moved. As a consequence of this, we propose that vestibular signals should be important to construct a sense of agency (the sense of being the agent of actions) and ownership of actions (“this action was *mine*”), two major constituents of self-consciousness (Jeannerod, 2006). Clinical observations revealed that vestibular signals are important to distinguish between self- and non-self motion, as patients with a vestibular loss are more likely to incorrectly interpret motion of objects or their environment as self-motion (e.g., Johnson et al., 1999). In addition, these patients may report not being in control of their self, as measured using depersonalization and derealization questionnaires (Yen Pik Sang et al., 2006).

In addition to distinguishing self- and non-self motion, we propose that vestibular signals are also important for constructing a more global sense of body ownership (“this body is *mine*”). Observations in neurological patients with somatoparaphrenia—who misattribute their own hand as belonging to someone else—are striking examples of relations between vestibular processing and body ownership. Caloric vestibular stimulation was showed to temporarily suppress somatoparaphrenia in these patients (Bisiach et al., 1991; Rode et al., 1992). In addition, experiments using the “rubber hand illusion” showed that galvanic vestibular stimulation increased illusory attribution of a non-corporeal hand under appropriate visuo-tactile conflicts (Lopez et al., 2010). Conclusively, these observations indicate that vestibular stimulation can interfere with the sensory and neural mechanisms of body ownership (see below) and modify the definition of self-other boundaries.

## Visual-vestibular interactions for the perception of bodies and emotions

Humans have evolved under a constant gravitational field. This invariant in the environment has strongly constrained the way bodies move and has moulded the development of the human sensorimotor system (Berthoz, 2000). Several studies have suggested the existence of an internal model of gravity in the human brain and highlighted the crucial role of the vestibular system in sensing gravity and constructing such model (McIntyre et al., 2001; Indovina et al., 2005). Interestingly, interpreting other's emotions and intentions requires subtle detection of other's body configurations (including facial expressions) and movements, a function for which the human visual system is finely tuned (Puce and Perrett, 2003; Troje et al., 2005). This knowledge appears fundamental for the survival of species as basic interactions necessitate detecting whether others have threatening posture and motion or aversive facial expressions. Several studies suggest that perception of faces and bodies depends on low-level visual mechanisms that are strongly orientation-dependent, i.e., depends on the visual stimuli orientation with respect to the observer's body and gravity. For example, Lobmaier and Mast (2007) showed that face perception depends on its orientation with respect to gravity. Lopez et al. (2009) showed that how we interpret the stability of whole-body postures is also a function of the gravitational reference. These data suggest that vestibular otolithic signals (sensing gravity) are used for the visual interpretation of socially relevant human body postures and kinematics and contribute to predict other's emotions and intentions.

## Third-person perspective taking and empathy

Another important aspect of social interaction is our ability to take another person's point of view—in other words, our ability to put oneself in someone else's shoes. This ability is referred to as “third-person perspective taking” in the literature and can be seen as the visuo-spatial ability through which one temporarily simulates the visual perspective of another individual (Vogeley and Fink, 2003). This is used, for example, to decide whether an object is on the right or left of someone (David et al., 2006; Lambrey et al., 2012). Psychophysical investigations have revealed that third-person perspective taking is rapid and involuntary (Tversky and Hard, 2009; Samson et al., 2010) and can be used to understand and predict feelings and intentions of others (Zwickel and Müller, 2010). Thus, some authors have drawn parallels between visuo-spatial perspective taking and empathy, another form of perspective taking allowing to understand emotional states of others (Berthoz, 2004; Mohr et al., 2010).

There is to date only few studies on the sensorimotor foundations of third-person perspective taking. As mentioned earlier, perspective taking necessitates the translocation of one's own egocentric viewpoint into a third-person, allocentric, reference. This operation requires geometrical transformations such as translations and rotations of the viewpoint. We propose that vestibular signals play an important role in these mental transformations of the viewpoint as they have been involved in several aspects of egocentric and allocentric mental imagery (Mast et al., 2006; Dilda et al., 2012). Indeed, stimulation of the semicircular canals during whole-body rotations on a chair modified performance in a whole-body mental transformation task (van Elk and Blanke, 2013). Similar disturbing effects of vestibular stimulation on whole-body mental imagery have been reported during galvanic (Lenggenhager et al., 2008) and caloric (Falconer and Mast, 2012) vestibular stimulation. In conclusion, we propose that vestibular signals are not only involved in self-motion perception, but also in mental simulation of self-motion, which seems necessary to adopt the (visual or affective) perspective of another individual.

## Self-other mirroring and the vestibular system

Social interactions involve the observation of other bodies in motion. There is now a large body of data showing that observing someone else's body can influence sensorimotor processing at the level of one's own body. Typical examples of mirroring between the self and others are contagious yawning and itching. Electrophysiological studies have revealed that self-other mirroring influences action execution: movement execution is facilitated by the observation of a body executing the same movement (Rizzolatti and Craighero, 2004). Similarly, the observation of someone else's face being touched facilitates the detection of tactile stimuli applied to one's own face (Serino et al., 2008). These effects have been related to a mirror neuron system, a group of neurons in the parieto-frontal cortex found crucial for social cognition (Rizzolatti and Craighero, 2004; Singer and Lamm, 2009). The mirror neuron system was also activated when observing another body performing complex actions (Calvo-Merino et al., 2005), being touched (Cardini et al., 2011) or experiencing pain (Singer et al., 2004).

Recently, Lopez et al. (2013) have speculated on the existence of a *vestibular mirror neuron system*. These authors showed that vestibular self-motion perception (measured on a whole-body motion platform imposing passive motions to the body) was influenced by the observation of videos showing passive whole-body motion of a body. In addition, this effect was correlated with scores of empathy: subjects that were the most empathic were more influenced by the observation of another body being moved passively. This study revealed a social influence on self-motion perception via vestibular processing. Accordingly, we suggest that how we experience our own body motion is constantly constrained by the motion of others around us. Further behavioral and neuroimaging studies should now be conducted to reveal the underlying neural mechanisms of these effects.

## Brain networks for vestibular processing and social cognition

**Figure 1:**
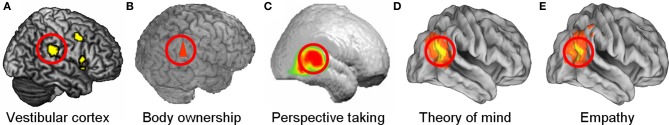
**Anatomical overlap between areas involved in vestibular processing, self-processing, and social cognition. (A)** Results of a meta-analysis showing brain regions activated by various techniques of vestibular stimulation. Adapted from Lopez et al. (2012). **(B)** Temporo-parietal cortex where transcranial magnetic stimulation modified illusory self-attribution of a non-corporeal object. Adapted from Tsakiris et al. (2008). **(C)** Temporo-parietal region involved in egocentric mental rotation tasks. Reproduced from Blanke et al. (2005). **(D,E)** Brain regions involved in theory of mind and empathy. Reproduced from Decety and Lamm (2007).

We propose that vestibular contributions to the sensorimotor mechanisms of social cognition are mediated by vestibular projections to multisensory regions found to be crucial for self and social processing. The vestibular cortex is composed of at least ten multisensory areas (review in Lopez and Blanke, 2011). This vestibular network is centered on the Sylvian fissure and covered the temporo-parietal junction (TPJ), superior temporal gyrus, inferior parietal lobule, parietal operculum, and insula (Figure 1A). Other important vestibular regions have been found in the primary and secondary somatosensory cortex, intraparietal sulcus, precuneus and cingulate cortex (Bottini et al., 1994; Dieterich et al., 2003; Kahane et al., 2003; Lopez et al., 2012).

Importantly, several vestibular areas overlap those classically found involved in social cognition. We propose that the TPJ, insula and cingulate cortex are the best candidates for vestibular–social interactions. Indeed, the right TPJ and posterior insula have been involved in the sense of owning a body: damage to the posterior insula distorts ownership for the left hand and may evoke the sensation that this hand belongs to someone else (i.e., somatoparaphrenia, Baier and Karnath, 2008). Self-other distinction also depends on multisensory processing in the TPJ as transcranial magnetic stimulation applied over the right TPJ modified illusory self-attribution of non-corporeal objects in the “rubber hand illusion” (Tsakiris et al., 2008) (Figure 1B).

Vestibular regions were also found activated in neuroimaging studies of third-person perspective taking (Figure 1C). These regions included the TPJ, intraparietal sulcus and precuneus (Vogeley et al., 2004; Blanke et al., 2005; David et al., 2006; Corradi-Dell'acqua et al., 2008; Kockler et al., 2010; Lambrey et al., 2012). The same regions are more generally involved in mental imagery (Zacks, 2008), suggesting that adopting someone else's viewpoint is a mental imagery process also requiring vestibular processing. Several studies have revealed that perspective taking shares functional and anatomical bases with mentalizing or theory of mind (e.g., Vogeley et al., 2001; Frith and Frith, 2003; Aichhorn et al., 2006). We note that these important aspects of social cognition activate several vestibular areas such as the TPJ and cingulate cortex (Figure 1D). Finally, neuroimaging studies of empathy also revealed an implication of vestibular regions such as the anterior insula, TPJ and cingulate cortex (Decety and Lamm, 2007; Bernhardt and Singer, 2012) (Figure 1E). Thus, understanding mental and bodily states of others may share mechanisms with adopting someone else's visuo-spatial perspective and require vestibular processing.

## Conclusion

We have summarized several behavioral and neuroimaging evidence from vestibular physiology and social neuroscience and have speculated on a vestibular contribution to several sensorimotor bases of social cognition. Yet, until now the vestibular cortex and neural bases of social cognition have been investigated in separate studies due to the lack of connections between the research fields of vestibular physiology and social neuroscience. We are optimistic that future research will endeavor to establish such interdisciplinary connections and propose that investigations of the multisensory foundations of social cognition should now incorporate the study of vestibular signals.
